# P-2039. Impact of Universal Decolonization in Intensive Care Units on Rates of Hospital-Acquired Staphylococcus aureus Infections

**DOI:** 10.1093/ofid/ofaf695.2203

**Published:** 2026-01-11

**Authors:** Charley A Nix, Kayla R Stover, Mary Joyce B Wingler, Katie Barber, Tulip A Jhaveri, David A Cretella

**Affiliations:** University of Mississippi Medical Center, Jackson, Mississippi; University of Mississippi School of Pharmacy, Jackson, MS; University of Mississippi Medical Center, Jackson, Mississippi; University of Mississippi, Jackson, MS; University of Mississippi Medical Center, Jackson, Mississippi; University of Mississippi Medical Center, Jackson, Mississippi

## Abstract

**Background:**

Decolonization strategies, such as universal decolonization, have been shown to be effective at reducing transmission and preventing infections in those colonized with Staphylococcus aureus. In 2023, the University of Mississippi Medical Center implemented universal decolonization in the intensive care units (ICU). The purpose of this study is to assess the effectiveness of this intervention on rates of hospital-acquired S. aureus infections.

**Methods:**

This single-center, pre- and post-intervention study evaluated patients admitted between May 1, 2022 to April 30, 2023 (pre-group) and September 11, 2023 to October 1, 2024 (post-group). The intervention consisted of five days of decolonization with twice-daily topical mupirocin ointment (2%) for all patients admitted to the ICU. Patients were included in the study if they were at least 18 years of age and had a hospital-acquired S. aureus bloodstream infection or pneumonia. Only the first incidence per patient per study period was included. The primary endpoint is the rate of hospital-acquired S. aureus infections (HAI-SA) before and after the implementation of universal decolonization. Secondary endpoints include the number of methicillin resistant S. aureus (MRSA) HAIs, methicillin-susceptible S. aureus (MSSA) HAIs, and all-cause in-hospital mortality.

**Results:**

A total of 452 patients were screened with 238 included. The median age of patients was 53.5 years, and the majority of patients were male (72%). In the post-group, 98.8% of patients were decolonized and the median time to de-colonization was 2 days. HAI-SAs declined from 158 patients to 80 patients, and the number of infections per 1000 patient days decreased from 5.36 to 2.47 in the pre- and post-group, respectively. More patients in the post-group had infections caused by MRSA (51% vs. 61%) compared with MSSA (49% vs. 39%) (p=0.144). In-hospital mortality was significantly lower in the post-group (32% vs 16%; p = 0.008).

**Conclusion:**

Universal decolonization reduced the incidence of HAI-SA in ICUs at an academic medical center and was associated with a significant decrease in all-cause in-hospital mortality.

**Disclosures:**

All Authors: No reported disclosures

